# Supplementary material for: The interactome of intact mitochondria by cross-linking mass spectrometry provides evidence for coexisting respiratory supercomplexes
Source: Mol Cell Proteomics. 2017 Dec 8;17(2):216–32. doi: 10.1074/mcp.RA117.000470 (PMC5795388; doi:10.1074/mcp.RA117.000470)
Supplement: Supplemental Data [file supp_RA117.000470_133922_0_supp_23978_fzffwf.zip › spectra_annotation/mito_spectra_annotation/461-1-9-1-4-1.pdf]

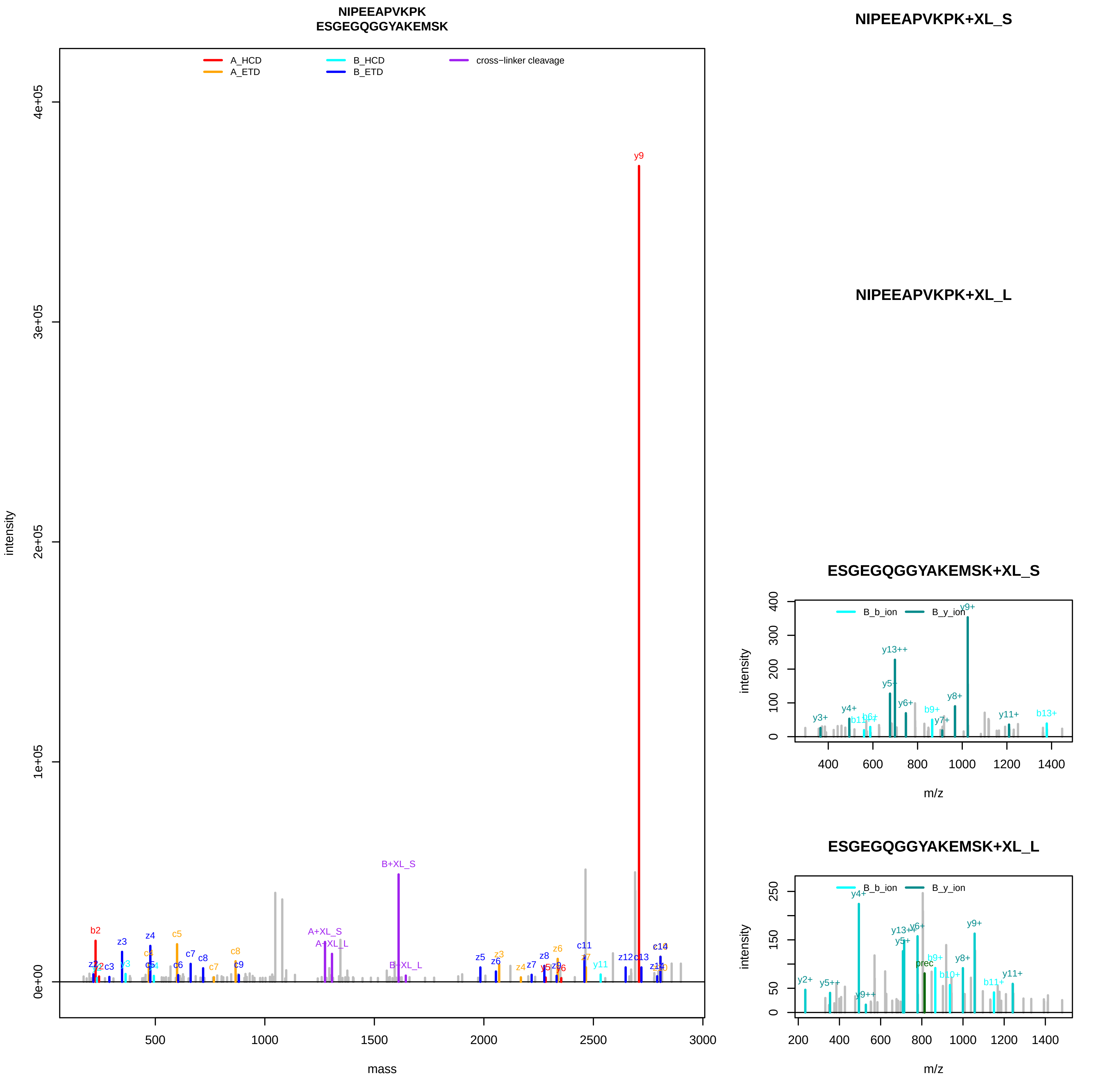
NIPEEAPVKPK
ESGEGQGGYAKEMSK
A_HCD
A_ETD
B_HCD
B_ETD
cross−linker cleavage
intensity
mass
y9
B+XL_S
A+XL_S
A+XL_L
B+XL_L
b2
z3
z4
c5
c7
c8
c9
c11
z12
c13
c14
y11
z5
z6
z7
z8
NIPEEAPVKPK+XL_S
NIPEEAPVKPK+XL_L
ESGEGQGGYAKEMSK+XL_S
B_b_ion
B_y_ion
y9+
y13++
y5+
y6+
y8+
b9+
y7+
y4+
y3+
y11+
b13+
m/z
ESGEGQGGYAKEMSK+XL_L
y4+
y6+
y13++
y9+
y8+
b9+
b10+
b11+
y11+
y2+
y5++
y9++
prec
